# Lessons Learned From a National Student-Led Audit by SPARC (Student Psychiatry Audit and Research Collaborative) on Emergency Department Management of Self-Harm in Under-18s

**DOI:** 10.1192/bjo.2025.10289

**Published:** 2025-06-20

**Authors:** Heather McAdam, Felicity Allman, Gloria Cheung, Ruth Goh, Samyak Pandey

**Affiliations:** 1 University of Glasgow, Glasgow, United Kingdom; 2 NHS Greater Glasgow and Clyde, Glasgow, United Kingdom; 3 Newcastle University, Newcastle, United Kingdom; 4 North Cumbria Integrated Care NHS Foundation Trust, Carlisle, United Kingdom; 5 York and Scarborough Teaching Hospitals, York, United Kingdom; 6 Imperial College London, London, United Kingdom; 7 Luton and Dunstable Hospital, Luton, United Kingdom

## Abstract

**Aims:** National audits provide valuable experience in research, leadership, and clinical governance. However, student-led initiatives present unique logistical and methodological challenges, including variability in training, data consistency, and long-term engagement. The Student Psychiatry Audit and Research Collaborative (SPARC) conducted a national audit to assess Emergency Department (ED) management of self-harm in under-18s across UK EDs between 2021 and 2023. This study aimed to examine the challenges encountered during the audit process and identify key lessons to inform future student-led research projects. Given the complexity of a multi-centre audit, we anticipated difficulties in training, data collection, and sustaining student engagement over two years.

**Methods:** Medical students were recruited as regional leads via university networks and psychiatric societies. A multidisciplinary committee of doctors and students oversaw the audit, which reviewed nearly 500 ED records across nine medical schools from 2021 to 2023. Data collection, based on National Institute for Health and Care Excellence (NICE) guidelines, involved a retrospective review but was complicated by a mid-audit guideline change, necessitating adaptations in data extraction. A snowball teaching method was used to train data collectors, who joined at different stages of the project. Following audit completion, challenges at each stage were analysed and compared with similar national audits to develop recommendations for future student-led initiatives.

**Results:** Nine medical schools participated, each led by a student regional coordinator responsible for local data collection, governance, and team management. However, 25% of records were excluded due to data quality issues, including errors in record eligibility and inconsistencies in questionnaire completion. The decentralised, peer-driven training model resulted in variable knowledge transfer, underscoring the need for structured training frameworks, clearer data verification processes, and automated data collection tools to improve consistency and accuracy.

**Conclusion:** This study highlights the importance of robust training and data management systems in student-led national audits. Key lessons include the need for structured protocols, ongoing data quality assessments, and strategies to maintain student engagement. Additionally, awareness of confounding factors such as regional variation and evolving clinical guidelines is crucial. These findings provide actionable recommendations to optimise future student-led clinical audits, promoting high-quality data collection and ensuring meaningful contributions to clinical governance.

